# Fungal Infection in Co-infected Patients With COVID-19: An Overview of Case Reports/Case Series and Systematic Review

**DOI:** 10.3389/fmicb.2022.888452

**Published:** 2022-07-06

**Authors:** Sima Sadat Seyedjavadi, Parmida Bagheri, Mohammad Javad Nasiri, Mehdi Razzaghi-Abyaneh, Mehdi Goudarzi

**Affiliations:** ^1^Department of Mycology, Pasteur Institute of Iran, Tehran, Iran; ^2^Department of Biotechnology, Faculty of Life Sciences and Biotechnology, Shahid Beheshti University, Tehran, Iran; ^3^Department of Microbiology, School of Medicine, Shahid Beheshti University of Medical Sciences, Tehran, Iran

**Keywords:** COVID-19, co-infection, fungal infection, systematic review, *Aspergillus*

## Abstract

Fungal co-infections are frequent in patients with coronavirus disease 2019 (COVID-19) and can affect patient outcomes and hamper therapeutic efforts. Nonetheless, few studies have investigated fungal co-infections in this population. This study was performed to assess the rate of fungal co-infection in patients with COVID-19 as a systematic review. EMBASE, MEDLINE, and Web of Science were searched considering broad-based search criteria associated with COVID-19 and fungal co-infection. We included case reports and case series studies, published in the English language from January 1, 2020 to November 30, 2021, that reported clinical features, diagnosis, and outcomes of fungal co-infection in patients with Severe Acute Respiratory Syndrome Coronavirus 2 (SARS-CoV-2). Totally, 54 case reports and 17 case series were identified, and 181 patients (132 men, 47 women, and 2 not mentioned) co-infected with COVID-19 and fungal infection enrolled. The frequency of fungal co-infection among patients with COVID-19 was 49.7, 23.2, 19.8, 6.6, and 0.5% in Asia, America, Europe, Africa, and Australia, respectively. Diabetes (59.6%) and hypertension (35.9%) were found as the most considered comorbidities in COVID-19 patients with fungal infections. These patients mainly suffered from fever (40.8%), cough (30.3%), and dyspnea (23.7%). The most frequent findings in the laboratory results of patients and increase in C-reactive protein (CRP) (33.1%) and ferritin (18.2%), and lymphopenia (16%) were reported. The most common etiological agents of fungal infections were *Aspergillus* spp., *Mucor* spp., *Rhizopus* spp., and *Candida* spp. reported in study patients. The mortality rate was 54.6%, and the rate of discharged patients was 45.3%. Remdesivir and voriconazole were the most commonly used antiviral and antifungal agents for the treatment of patients. The global prevalence of COVID-19-related deaths is 6.6%. Our results showed that 54.6% of COVID-19 patients with fungal co-infections died. Thus, this study indicated that fungal co-infection and COVID-19 could increase mortality. Targeted policies should be considered to address this raised risk in the current pandemic. In addition, fungal infections are sometimes diagnosed late in patients with COVID-19, and the severity of the disease worsens, especially in patients with underlying conditions. Therefore, patients with fungal infections should be screened regularly during the COVID-19 pandemic to prevent the spread of the COVID-19 patients with fungal co-infection.

## Introduction

Severe Acute Respiratory Syndrome Coronavirus 2 (SARS-CoV-2) causes coronavirus disease 2019 (COVID-19) that started as a local epidemic but evolved within a few months into a worldwide pandemic with high morbidity and mortality rates, and the World Health Organization declared it as a global epidemic on January 30, 2020 (Dos Santos et al., 2020; Gorbalenya et al., 2020). The prognosis of this disease is severe in patients with underlying conditions. Diabetes, hypertension, cancer, chronic kidney disease, heart failure, and mental disorders increased mortality. However, success in developing specific therapeutic against COVID-19 infection is still needed (Robinson et al., 2022). Therefore, the most effective way to deal with an epidemic is to prevent further infection. The elevated prevalence of mortality and infection in patients with COVID-19 can be due to natural immunity and replication of the virus in the lower respiratory tract, and also due to superinfections and secondary infections, resulting in severe lung damage as well as acute respiratory distress syndrome (ARDS) (Zheng et al., 2003; Farhan et al., 2021). Patients with COVID-19 are found with co-infections with respiratory viruses, bacteria, fungi, and secondary infections that have been identified as a fatal predictor. From the outbreak of COVID-19, we found that fungal co-infection of patients with COVID-19 could significantly increase mortality rates (Yang S. et al., 2021). The significance of fungal co-infection in patients with COVID-19, however, especially in patients with severe and critical conditions, is still poorly understood (Yang et al., 2020). Invasive fungal infections, including aspergillosis and candidiasis, are frequent in hospitalized patients (Sadeghi et al., 2018; Jamzivar et al., 2019; Hughes et al., 2020; Nasir et al., 2020). Acute respiratory diseases, such as invasive pulmonary aspergillosis (IPA), are common in intensive care units (ICUs) and immunocompromised patients (Prattes et al., 2021). Fungal infections, before or after COVID-19, are capable of complicating COVID-19 diagnosis, treatment, and progression (Talento and Hoenigl, 2020). According to data obtained from other COVID-19 outbreaks [severe acute respiratory syndrome (SARS) and the Middle East respiratory syndrome (MERS)], invasive aspergillosis and also other systemic fungal infections play a role in severe outcomes of patients in ICUs (Song et al., 2020). In addition, patients with COVID-19 with predisposing factors (mechanical ventilation, diabetes, and cytokine storm) were found with a dramatic increase in the incidence of opportunistic fungal infections (Silva et al., 2020; Song et al., 2020). In contrast, because of the complicated medical situations of the patients with COVID-19 and the improper collection of the clinical species, many fungal infections in these patients are misidentified (Silva et al., 2020). Researchers are facing several challenges in the diagnosis and identification of fungal infections. In this systematic review, we reviewed the case reports and case series with patients with COVID-19 presenting fungal co-infections to evaluate the various aspects such as symptoms, diagnosis, and the most frequent etiological agents of patients with fungal co-infecting COVID-19, treatment, and outcome.

## Materials and Methods

### Search Strategy

A comprehensive systematic literature search was conducted by reviewing original research papers published in Medline, Web of Science, and Embase databases. The following keywords were used for the search: “coronavirus,” “coronavirus infections,” “HCoV,” “nCoV,” “Covid,” “SARS,” “COVID-19,” “nCoV19,” “SARS-CoV-2,” “SARS coronavirus 2,” “2019 novel corona virus,” “Human,” “pneumonia,” “SARS,” “co-infection,” “Superinfection,” “fungus,” “mycosis,” “co-infect,” “secondary infection,” “mixed infection,” “Fungal infection,” “aspergillosis,” “CAPA,” and “upper respiratory” alone or in combination with “OR” and/or “AND.” The search included English language studies from January 1, 2020 to November 30, 2021. Then, articles were kept if the title and abstract contained discussion about bacterial, fungal, and/or respiratory viral co-infection in patients with SARS-CoV-2. The systematic review was performed based on the Preferred Reporting Items for Systematic Reviews and Meta-Analyses (PRISMA) instructions (Moher et al., 2010).

### Ethical Statement

As this study was a systematic review, it did not require any ethics committee approval.

### Inclusion and Exclusion Criteria

All case reports/case series that were about the fungal infection among patients with COVID-19 in English were evaluated. They included adequate data for analysis, namely, country of origin, the number of patients with COVID-19, and the number of cases with fungal infections, fungal species/group, clinical signs, laboratory results, diagnostic techniques, outcomes, and treatment.

The following exclusion criteria were used: (1) only animal studies, (2) research on fungal infections only, (3) research on patients with COVID-19 only, (4) review articles, (5) meeting or congress abstracts, (6) editorials, (7) letters, (8) languages other than English, (9) meta-analyses or systematic reviews, (10) articles available only in abstract, and (11) duplicate studies.

### Study Selection and Data Extraction

The obtained studies were merged, followed by removing the duplicates using EndNote X7 (Thomson Reuters, United States). Two authors (PB and MG) separately screened the studies according to their titles and abstracts, considering the exclusion and inclusion criteria of this study. The full texts were analyzed by a third author (SS). Data extracted included the first author’s last name, research type, publication year, country, number of patients with COVID-19, number of cases with fungal confection, co-infecting fungi, clinical symptoms, laboratory findings and outcomes, diagnostic methods, and treatment. The data were obtained by two independent individuals and validated by another investigator.

### Quality Assessment

Quality assessment was performed by a checklist provided by the Joanna Briggs Institute (JBI).

## Results

### Characteristics of the Selected Studies

**FIGURE 1:**
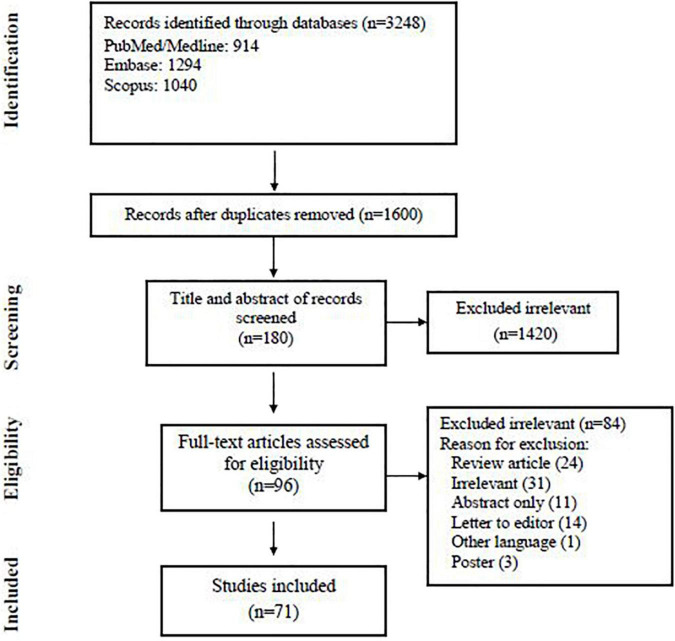
Flowchart of study selection for inclusion in the systematic review and meta-analysis.

Our search yielded 3,248 records from three databases; we excluded 1,648 duplicates and screened 1,600 articles. At the abstract and title review stage, we excluded 1,420 articles, leaving 180 articles for full-text review. After reviewing the full text of 180 studies, eventually, 71 articles met the inclusion criteria and were subjected to the final assessment (Figure 1). Table 1 summarizes the characteristics of published data related to fungal co-infection in patients with COVID-19.

**TABLE 1:** Characteristics of included prevalence studies.

References	Published time	Country	Type of study	Patients with COVID-19	Patients with co-infection	Diagnostic method of COVID-19	Diagnostic method of fungi	Fungi	Mean age	Male/Female
Rubiano et al., 2021	2020	United States	Case report	1	1	PCR	Direct immunofluorescence and PCR tests of BAL	*Pneumocystis jirovecii*	36	1M
Werthman-Ehrenreich, 2021	2020	United States	Case report	1	1	PCR	Cultures of sinus	*Mucor* spp.	33	1F
Chang et al., 2020	2020	United States	Case report	1	1	nr	Serologic tests	*Coccidioides* spp.	48	1F
Shah et al., 2020	2020	United States	Case report	1	1	nr	Serologic tests	*Coccidioides* spp.	48	1M
Mekonnen et al., 2021	2021	United States	Case report	1	1	nr	Histopathology examination and fungal culture	*Rhizopus* spp.	60	1M
De Francesco et al., 2020	2020	Italy	Case report	1	1	RT-PCR	Culture and RT-PCR of sputum	*Aspergillus fumigatus* and *Pneumocystis jirovecii*	65	1F
Meijer et al., 2020	2020	Netherlands	Case report	1	1	PCR	Culture of TA, detection of GM in TA, and BDG in serum	*Aspergillus fumigatus* (triazole-resistant)	74	1F
Passarelli et al., 2020	2020	Brazil	Case report	1	1	RT-PCR	Blood culture	*Cryptococcus neoformans*	75	1M
Posteraro et al., 2020	2020	Italy	Case report	1	1	RT-PCR	Blood culture and MALDI-TOF	*Candida glabrata* (resistant all echinocandins)	79	1M
Seitz et al., 2020	2020	Austria	Case report	1	1	PCR	Culture of the removed central venous catheter	*Candida glabrata*	72	1M
Ventoulis et al., 2020	2020	Greece	Case report	2	2	RT-PCR	Blood cultures, direct microscopy, germ tube testing, biochemical testing, molecularly and sequencing	*Saccharomyces cerevisiae*	76–73	2M
Do Monte Junior et al., 2020	2020	Brazil	Case report	1	1	PCR	Pathological examination	*Mucor* spp.	86	1M
Schein et al., 2020	2020	France	Case report	1	1	RT-PCR	Detection of GM in sputum and blood, serology, serum PCR	*Aspergillus* spp.	87	1F
Mehta and Pandey, 2020	2020	India	Case report	1	1	RT-PCR	Nasal biopsy and culture	*Mucor* spp.	60	1M
Pasero et al., 2021	2021	Italy	Case report	1	1	RT-PCR	Bronchial aspirate culture, microbiological and histopathological examination	*Candida glabrata* and *Rhizopus* spp.	66	1M
Nasri et al., 2020	2020	Iran	Case report s	1	1	RT-PCR	Detection of GM in Serum	*Aspergillus* spp.	42	1F
Placik et al., 2020	2020	United States	Case report	1	1	RT-PCR	Microbiological analysis of the intraoperative specimens	*Rhizopus* spp.	49	1M
Prattes et al., 2021	2021	Austria	Case report	1	1	PCR	ETA culture, Aspergillus lateral-flow device (LFD) in ETA	*Aspergillus fumigatus*	70	1M
Ghelfenstein-Ferreira et al., 2021	2021	France	Case report	1	1	RT-PCR	TA culture, quantitative PCR	*Aspergillus fumigatus*	56	1M
Abdalla et al., 2020	2020	Qatar	Case report	2	2	PCR	Lower respiratory culture	*Aspergillus niger* (1/2), *Aspergillus terreus* (1/2), *Candida albicans* (2/2)	66	2M
Dos Santos et al., 2020	2020	Brazil	Case report	1	1	RT-PCR	Tongue scrape culture	*Saccharomyces cerevisiae*	67	1M
Sharma et al., 2021	2021	Australia	Case report	1	1	RT-PCR	ETA culture	*Aspergillus fumigatus*	66	1F
Antinori et al., 2020	2020	Italy	Case report	1	1	RT-PCR	BAL culture, detection of GM in serum	*Aspergillus fumigatus*	73	1M
Al Osta et al., 2021	2021	Lebanon	Case report	1	1	nr	Microscopic examination of the palate biopsy	*Mucor* spp.	62	1M
Khatri et al., 2021	2021	United States	Case report	1	1	RT-PCR	Culture of aspirate fluid along the anterior right upper chest wall	*Rhizopus microsporus*	68	1M
Fernandez et al., 2021	2021	Argentina	Case report	1	1	Molecular testing	MALDI-TOF in TA and detection of GM in serum	*Aspergillus flavus* and *Candida lusitaniae*	85	1M
Haglund et al., 2021	2021	Denmark	Case report	1	1	RT-PCR	Morphological analysis, detection of GM, MALDI-TOF, and PCR of BAL	*Aspergillus fumigatus*	52	1M
Mohamed et al., 2021	2021	Ireland	Case report	1	1	RT-PCR	Culture of ETA, detection of BDG in serum and GM in ETA and serum	*Aspergillus fumigatus* (triazole resistance) and *Candida albicans*	55	1M
Dallalzadeh et al., 2021	2021	United States	Case report	2	2	Rapid PCR	Culture of purulence from the eye and MRI of the sinonasal cavity	*Rhizopus* spp. and *Mucor* spp.	38–46	2M
Merchant et al., 2021	2021	United States	Case report	1	1	PCR	Immunofluorescence of BAL	*Pneumocystis jirovecii*	38	1M
Trovato et al., 2021	2021	Italy	Case report	1	1	PCR	Detection of BDG and GM in serum, microscopic and culture, MALDI-TOF and RT-PCR of bronchoaspirate sample	*Aspergillus niger*	73	1M
Waizel-Haiat et al., 2021	2021	Mexico	Case report	1	1	RT-PCR	Direct exam and culture	*Lichteimia* (*Absidia*) spp.	26	1F
Basso et al., 2021	2021	Brazil	Case report	1	1	PCR	Sputum microscopy and detection of *Histoplasma capsulatum* antigen in the urine sample	*Histoplasma capsulatum*	43	1F
Viceconte et al., 2021	2021	Italy	Case report	1	1	PCR	Direct immunofluorescence of BAL	*Pneumocystis jirovecii*	50	1M
Aldaas et al., 2021	2021	United States	Case report	1	1	RT-PCR	Chest CT, detection of GM in BAL	*Aspergillus* spp.	72	1M
Khodavaisy et al., 2021	2021	Iran	Case report	1	1	RT-PCR	Fungal DNA extraction	*Aspergillus tubingensis*	59	1M
Maini et al., 2021	2021	India	Case report	1	1	RT-PCR	Microbiological studies on tissue biopsies (positive PAS and GMS)	*Rhizopus oryzae*	38	1M
Saldanha et al., 2021	2021	India	Case report	1	1	Molecular testing	Histopathological examination	*Mucor* spp.	32	1F
Legnani and Dusi, 2021	2021	Italy	Case report	1	1	RT-PCR	BAL culture, quantitative PCR	*Aspergillus fumigatus* and *Rhizopus microsporus*	55	1M
Chaudhary et al., 2022	2021	India	Case report	1	1	RT-PCR	Pus analysis	*Mucor* spp.	21	1M
Alekseyev et al., 2021	2021	United States	Case report	1	1	RT-PCR	Right sphenoid sinus secretions culture	*Mucor* spp.	41	1M
Kanwar et al., 2021	2021	United States	Case report	1	1	PCR	Sputum culture, MALDI-TOF, Sequence analysis	*Rhizopus azygosporus*	56	1M
Revannavar et al., 2021	2021	India	Case report	1	1	RT-PCR	Histopathological analysis and fungal culture	*Rhizopus* spp.	NM	1F
Ali et al., 2021	2021	Qatar	Case report	1	1	PCR	Blood Culture, MALDI-TOF	*Trichosporon asahii*	58	1M
Khan et al., 2020	2021	United States	Case report	1	1	RT-PCR	ETA culture and biopsy and BAL	*Aspergillus flavus*, *Aspergillus niger*, *Candida albicans*, *Candida glabrata*, *Candida krusei*	44	1F
Veisi et al., 2021	2021	Iran	Case report	2	2	PCR	Histopathological examinations	*Mucor* spp.	46	1F/1M
Imoto et al., 2021	2021	Japan	Case report	1	1	Molecular testing	sputum culture, detection of GM and BDG in serum	*Aspergillus fumigatus*	72	1M
Arana et al., 2021	2021	Spain	Case report	2	2	nr	Debridement culture, culture from necrotic tissue, palate biopsy	*Rhizopus oryzae*, *Mucor* spp.	55	2M
Ohashi et al., 2021	2021	Japan	Case report	1	1	PCR	Oral swab culture	*Candida albicans*	75	1M
Johnson et al., 2021	2021	United States	Case report	1	1	PCR	BAL culture, detection of GM and BDG in serum	*Aspergillus fumigatus* and *Rhizopus arrhizus*	79	1M
Sari et al., 2021	2021	Indonesia	Case report	1	1	RT-PCR	Blood culture	*Candida tropicalis*	54	1F
Alobaid et al., 2021	2021	Kuwait	Case report	2	2	RT-PCR	BAL and ETA culture	*Aspergillus niger* (2/2)	NM	NM
Costache et al., 2021	2021	Romania	Case report	1	1	RT-PCR	Microbiologic examination of sputum sample	*Aspergillus flavus* and *Aspergillus fumigatus*	53	1F
Mehrabi et al., 2021	2021	Iran	Case report	1	1	RT-PCR	Pathology evaluation of the paranasal sinus tissue	*Mucor* spp.	51	1M
Mitaka et al., 2020	2020	United States	Case series	4	4	RT-PCR	Respiratory cultures	*Aspergillus fumigatus*	79	4M
Benedetti et al., 2021	2020	Argentina	Case series	5	5	RT-PCR	Detection of GM in serum and respiratory samples, cultures of sputum, tracheal aspirate	*Aspergillus fumigatus* (5/5), *Candida albicans* (1/5)	52.4	1F/4M
Wang et al., 2020	2020	China	Case series	8	8	nr	Sputum or BAL culture	*Aspergillus fumigatus* (8/8)	73	8M
Lescure et al., 2020	2020	France	Case series	5	1	RT-PCR	Tracheal aspirates culture	*Aspergillus flavus*	47	2F/3M
Falces-Romero et al., 2020	2020	Spain	Case series	10	10	RT-PCR	Sputum and BAL culture	*Aspergillus fumigatus* (9/10) and *Aspergillus nidulans* (1/10)	69.5	2F/8M
Martins et al., 2021	2021	Brazil	Case series	8	8	PCR	Non-bronchoscopic lavage and blood culture	*Aspergillus flavus* (1/8), *Aspergillus fumigatus* (3/8), *Candida orthopsilosis* (1/8), *Candida albicans* (1/8), *Candida krusei* (1/8), *Candida lusitaniae* (1/8), *Cryptococcus neoformans* (1/8)	66	3F/5M
Singh V. et al., 2021	2021	India	Case series	10	10	RT-PCR	Nasal tissue culture	*Aspergillus flavus* (7/10), *Aspergillus fumigatus* (3/10), *Rhizopus arrhizus* (7/10)	49.2	4F/6M
Almeida et al., 2021	2021	Brazil	Case series	2	2	RT-PCR	MALDI-TOF, sequencing	*Candida auris* (2/2)	65.5	1F/1M
Kalpana et al., 2021	2021	India	Case series	15	15	nr	Histopathological examination	*Mucor* spp.	NM	2F/13M
Singh V. et al., 2021	2021	India	Case series	13	13	RT-PCR	Positive KOH mount, clinical features	*Mucor* spp.	38	3F/10M
Nehara et al., 2021	2021	India	Case series	5	5	RT-PCR	Histopathological examination, culture of the sinonasal specimen	*Mucor* spp. (5/5)	62.2	4F/1M
Bowalekar et al., 2021	2021	India	Case series	10	10	PCR	Culture	*Aspergillus flavus* (2/10), *Aspergillus fumigatus* (2/10), *Rhizopus arrhizus* (6/10)	55.4	4F/6M
Mishra et al., 2021	2021	India	Case series	10	10	nr	Histopathological examination	*Mucor* spp. (10/10)	55.8	1F/9M
Teixeira et al., 2021	2021	Brazil	Case series	4	2	RT-PCR	Urine culture	*Candida albicans* (2/2)	68.75	3F/1M
Ashour et al., 2021	2021	Egypt	Case series	8	8	RT-PCR	Histopathology and culture	*Aspergillus* spp. (1/8), *Mucor* spp. (6/8)	53.62	3F/5M
Roushdy and Hamid, 2021	2021	Egypt	Case series	4	4	PCR	Pathological assessment	*Mucor* spp. (4/4)	67.75	1F/3M
Flikweert et al., 2020	2020	Netherlands	Case series	7	6	RT-PCR	Clinical, radiological, and mycological data, detection of GM in serum, sputum and BAL, tracheal or bronchial culture, ELISA is used for GM detection	*Aspergillus fumigatus*	74	2F/5M

*RT-PCR, real time-polymerase chain reaction; MALDI-TOF, matrix-assisted laser desorption/ionization-time of flight; TA, tracheal aspirate; ETA, endotracheal aspirate; BDG, 1–3, β-D-glucan; GM, galactomannan; BAL, bronchoalveolar lavage; nr, not reported.*

### The Frequency of Fungal Infections Among Patients With COVID-19

The characteristics of the 71 included articles are shown in Table 2. Fifty-four case reports and seventeen case series highlighted fungal co-infection in 60 and 121 patients with COVID-19, respectively. Conforming to the results of these studies, 181 patients with fungal infections had been declared among 188 patients with COVID-19 from 23 countries (Table 2). Based on the data in this table, most of the patients in this study were reported from India (68 patients), United States (19 patients), Brazil (18 patients), and Spain/Egypt (12 patients for each), respectively. Among the cases with defined gender, 47 cases with fungal infections were women and 132 were men. The rate of co-infection in the age group of less than 50 years and more than 50 years was 23.7 and 66.2%, respectively. Table 3 shows more details of the subgroup analysis of the studies.

**TABLE 2:** Frequency of fungal co-infection among patients with COVID-19 based on different subgroups.

Types of study	Number of studies	Number of patients with COVID-19	Number of patients with fungal co-infection	%
**Case report**	54	60	60	100
**Case series**	17	128	121	94.53
**Continent**	**Variables**	**Number of patients with fungal co-infection**	*n*/N*	**%**
	America	42	42/181	23.2
	Asia	90	90/181	49.7
	Europe	36	36/181	19.8
	Australia	1	1/181	0.55
	Africa	12	12/181	6.6
**Gender**	Male	132	132/181	72.9
	Female	47	47/181	25.9
	nr	2	2/181	1.1
**Age**	Less than 50 years	43	43/181	23.7
	More than 50 years	120	122/181	66.2
	nr	18	18/181	9.9

*n, number of patients with any variable; N, total number of COVID-19 patients with fungal co-infections.

**TABLE 3:** Summary of the case reports/case series findings.

Comorbidities	Variables	Number of patients with fungal co-infection	*n/N* *	%
	Obesity	15	15/181	8.2
	Hyperlipidemia	7	7/181	3.8
	Hypertension	65	65/181	35.9
	Diabetes	108	108/181	59.6
	Ischemic disease	8	8/181	4.4
	Metabolic acidosis	4	4/181	2.2
	Diabetes ketosis	4	4/181	2.2
	Smoker	10	10/181	5.5
	HIV	3	3/181	1.6
	Urinary tract infection	3	3/181	1.6
	Atrial fibrillation	4	4/181	2.2
	Kidney transplantation	5	5/181	2.7
	Heart transplantation	2	2/181	1.1
	Heart disease	2	2/181	1.1
	Depression	1	1/181	0.55
	Kidney injury	9	9/181	4.9
	Chronic liver disease	1	1/181	0.55
	Liver cirrhosis	1	1/181	0.55
	Renal failure	8	8/181	4.4
Clinical manifestation	Cough	55	55/181	30.3
	Fever	74	74/181	40.8
	Nausea	2	2/181	1.1
	Dyspnea	43	43/181	23.7
	Tachypnea	18	18/181	9.9
	Vomiting	5	5/181	2.7
	Fatigue	7	7/181	3.8
	Tachycardia	11	11/181	6
	Headache	21	21/181	11.6
	Chest pain	2	2/181	1.1
	Diarrhea	15	15/181	8.2
	Shortness of breath	23	23/181	12.7
	Malaise	4	4/181	2.2
	Sinus congestion	4	4/181	2.2
	Body ache	3	3/181	1.6
	Muscle ache	2	2/181	1.1
	Abdominal pain	3	3/181	1.6
	Chills	2	2/181	1.1%
	Sore throat	4	4/181	2.2
Fungal infections evidences in patients with COVID-19	Pulmonary embolism	4	4/181	2.2
	Proptosis	15	15/181	8.2
	Conjunctival chemosis	8	8/181	4.4
	Periorbital edema	11	11/181	6
	Facial swelling and sinusitis	13	13/181	7.1
	Sternal wound	1	1/181	0.55
	Encephalopathy	1	1/181	0.55
	Lid swelling and maxillary	4	4/181	2.2
	Soft tissue edema	4	4/181	2.2
	Ophthalmoplegia	14	14/181	7.7
	Dry skin and mucus	6	6/181	3.3
	Cerebral hemorrhage	2	2/181	1.1
	Renal failure	7	7/181	3.8
	Multi organ failure	12	12/181	6.6
	Sepsis shock	14	14/181	7.7
	Respiratory failure	9	9/181	4.9
Laboratory findings	Leukopenia	3	3/181	1.6
	Lymphopenia	29	29/181	16
	Leukocytosis	22	22/181	12.1
	High ferritin	33	33/181	18.2
	High pro-calcitonin	20	20/181	11
	Low albumin	17	17/181	9.3
	Thrombocytopenia	5	5/181	2.7
	High C-reactive protein	60	60/181	33.1
	High D-dimer	24	24/181	13.2
Chest CT scan	ground-glass opacity	46	46/181	25.4
	bilateral infiltrates	36	36/181	19.8
Outcome	Death	101	99/181	54.6
	Recovered	81	82/181	45.3
	nr	12	12/181	6.6

*n, number of patients with a specific variable; N, total number of COVID-19 patients with fungal co-infections; nr, not reported.

Among 19 types of comorbidities, diabetes (59.6%), hypertension (35.9%), and obesity (8.2%) were the commonest comorbidities. Fever (40.8%), cough (30.3%), dyspnea (23.7%), and shortness of breath (12.7%) were the commonest clinical symptoms in COVID-19 patients with fungal infections. Laboratory assessment of patients indicated that elevated C-reactive protein (CRP) (>100 mg/L) (33.1%), high ferritin (>500 ng/mL) (18.2%), lymphopenia (<800 cells/μl) (16%), leukocytosis, and increased D-dimer (>1,000 ng/ml) (13.2%) were the most common findings (Table 3).

Computerized tomography (CT) scan has been reported in studies as a diagnostic method employed for COVID-19, and its findings are as follows: ground-glass opacification (25.4%) and bilateral infiltrates (19.8%). The CT results in the majority of the assessed patients were ground-glass opacification. We also considered the patients’ outcomes, and of 181 patients (mentioned in Table 2), 81 improved, 101 died, and in 12 patients, the outcome was unknown (Table 3).

According to the results of this study (Table 4), RT-PCR was the most common laboratory technique for the detection of SARS-CoV-2 in the study patients (43 articles). The most frequently used laboratory techniques for co-fungal detection within studies included 52 that used culture, 13 that used galactomannan (GM) and/or 1,3 β-D-glucan (BDG) detection test, 14 that used histopathology examination, and 14 that used matrix-assisted laser desorption ionization time of flight (MALDI-TOF) and/or molecular detection.

**TABLE 4:** Diagnostic methods for patients with COVID-19 and fungal infection.

COVID-19 detection	Variables	Number of studies
	RT-PCR	40
	PCR	20
	Molecular testing	3
	nr	8
Fungal detection	Culture	52
	Detection of GM and/or BDG	13
	Pneumocystis antigen detection	3
	MALDI-TOF and/or molecular detection	14
	Histopathology examination	14
	Serologic tests	3

*RT-PCR, real time-polymerase chain reaction; PCR, polymerase chain reaction; MALDI-TOF, matrix-assisted laser desorption/ionization-time of flight; BDG, 1–3, β-D-glucan; GM, galactomannan; nr, not reported.*

From the fungal co-infections registered, the most common etiological agents were as follows: *Aspergillus* spp. (82 isolates), *Mucor* spp. (69 isolates), *Rhizopus* spp. (24 isolates), *Candida* spp. (21 isolates), *Pneumocystis jirovecii* (four isolates), *Saccharomyces cerevisiae* (three isolates), *Coccidioides* spp. and *Cryptococcus neoformans* (two for each), *Trichosporon asahii* (six isolates), and *Histoplasma capsulatum* and *Lichteimia* (*Absidia*) (one for each) were infections in patients with fungal-COVID-19 (Table 5). In the study articles, the drugs applied to treat COVID-19 patients with fungal infections were characterized into three categories, namely, antibacterial, antiviral, and antifungal drugs (Table 6). Remdesivir (45.74%) and lopinavir/ritonavir (12%) were the most common antiviral drugs used. Among the antifungal drugs reported in Table 6, amphotericin B (50%) and voriconazole (22.16%) were widely used as an antifungal agent. Among the antifungal drugs reported in Table 6, amphotericin B (50%) and voriconazole (22.16%) were the most widely used antifungal agents for treating patients.

**TABLE 5:** Fungal pathogens detected in patients with COVID-19.

Fungal type	Fungal genera	Fungal species	Number of isolates
	*Candida*	*Candida albicans*	9
		*Candida glabrata*	2
		*Candida glabrata* (all echinocandins resistant)	2
		*Candida lusitaniae*	2
		*Candida tropicalis*	1
		*Candida krusei*	2
		*Candida auris*	2
		*Candida orthopsilosis*	1
	*Aspergillus*	*Aspergillus* spp.	4
		*Aspergillus fumigatus*	50
		*Aspergillus flavus*	18
		*Aspergillus fumigatus* (triazole-resistant)	2
		*Aspergillus niger*	5
		*Aspergillus terreus*	1
		*Aspergillus tubingensis*	1
		*Aspergillus nidulans*	1
	*Pneumocystis*	*Pneumocystis jirovecii*	4
	*Histoplasma*	*Histoplasma capsulatum*	1
	*Rhizopus*	*Rhizopus microsporus*	2
		*Rhizopus* spp.	5
		*Rhizopus arrhizus*	14
		*Rhizopus oryzae*	2
		*Rhizopus azygosporus*	1
	*Saccharomyces*	*Saccharomyces cerevisiae*	3
	*Cryptococcus*	*Cryptococcus neoformans*	2
	*Coccidioides*	*Coccidioides* spp.	2
	*Lichteimia*	*Lichteimia* (*Absidia*) spp.	1
	*Mucor*	*Mucor* spp.	69
	*Trichosporon*	*Trichosporon asahii*	1

**TABLE 6:** Agents used in the treatment of patients with fungal co-infection.

Antiviral drug	Agent	Number of patients with co-infection	n/N* (%)
	Remdesivir	43	43/94(45.74)
	Lopinavir/ritonavir	12	12/94(12.76)
	Oseltamivir	7	7/94(7.44)
	Darunavir/ritonavir	3	3/94(3.2)
	Hydroxychloroquine	27	27/94(28.72)
	Dolutegravir/emtricitabine/ tenofovir alafenamide	1	1/94(1.06)
	Bictegravir/emtricitabine/ tenofovir alafenamide	1	1/94(1.06)
Antibacterial drug	Antibacterial drug	82	82/108(75.9)
	Azithromycin	26	26/108(24.1)
Antifungal drugs	Amphotericin B	111	111/185(60)
	Anidulafungin	8	8/185(4.3)
	Voriconazole	41	41/185(22.16)
	Isavuconazole	6	6/185(3.2)
	Micafungin	6	6/185(3.2)
	Fluconazole	10	10/185(5.4)
	Caspofungin	9	9/185(4.8)
	Itraconazole	3	3/185(1.6)

*n, number of patients with any variable; N, total number of COVID-19 patients with fungal co-infections.

## Discussion

This systematic review is a detailed description of fungal co-infections in patients with COVID-19. There is a special concern for fungal infections, before or after COVID-19 exposure, which leads to treatment failure and deterioration of disease and imposes high healthcare costs on patients and hospitals. Overall, it is well established that all genders and ages are at risk for COVID-19 infection (Kalantari et al., 2020; Song et al., 2020; Talento and Hoenigl, 2020).

In this systematic review, we analyzed 181 fungal patients with COVID-19 from 23 countries, and co-infection in the age group of over 50 years was higher than under 50 years (66.2 vs. 23.7%) which is in agreement with studies that exhibited elderly patients have a higher risk of COVID-19 infection and mortality. Our data are in concordance with a study conducted in the United Kingdom on co-infection patients with COVID-19 symptoms, which reported that the highest prevalence of co-infection patients was in the age group of 55–81 years (Hughes et al., 2020). In this connection, Senok et al. (2021) found that the mean age of patients with co-infections was 49.3 ± 12.5 years in the United Arab Emirates. These observations indicated that declined immune system ability and increasing comorbid conditions with age could be a rational justification for the observed increased infection in older patients. The patients’ gender was assessed in 71 studies that indicated COVID-19 infection in men (72.9%) was higher than that of women (25.9%). A research performed by Senok et al. (2021) on patients hospitalized with COVID-19 in the United Arab Emirates notified that the most cases (84.2%) were men. Garcia-Vidal et al. (2021) found that the majority of patients hospitalized with COVID-19 in Spain were in the age of 62 years and also the most cases (55.8%) were men. In a single-center experiment performed by Jin et al. (2020), in China, out of 43 patients with COVID-19, 51.2% were found to be men. As a finding, it can be inferred that sex hormones and X chromosomes as factors involved in innate and adaptive immunity may have an important role in less susceptibility to COVID-19 infection among women. Overall, the high occurrence of many diseases in men compared to women could likely indicate a shorter life expectancy in this sex. Consequently, gender would be considered a risk factor for higher morbidity and severity in patients with COVID-19.

The disease pattern of COVID-19 can range from mild to life-threatening pneumonia associated with bacterial and fungal co-infections (Mehta and Pandey, 2020). Due to the associated comorbidities [e.g., diabetes mellitus, hypertension, and chronic obstructive pulmonary disease (COPD)] and immunocompromised conditions, these patients are prone to develop severe opportunistic infections. The findings of this study indicated that diabetes, hypertension, and obesity were the most common comorbidities reported in patients with fungal co-infections and COVID-19. This result is in line with the results of Abdalla et al. (2020) which indicated that diabetes, hepatitis B, and hypertension are the common comorbidities in patients with COVID-19-associated pulmonary aspergillosis. Other reports showed that in a patient with diabetes and leukemia, *Aspergillus fumigatus* was isolated from BAL (Dallalzadeh et al., 2021). Published data have indicated that obesity is a risk factor for infection with COVID-19 (Albashir, 2020; Yang J. et al., 2021). Based on the evidence, the relationship between inflammation and hypertension is well documented. Patients with inflammatory responses increase the disease’s severity and complications, which make the infection worse. In line with our report, Mirzaei et al. (2021) in their review reported diabetes, obesity, and COPD as the most common underlying diseases in patients with COVID-19. Underlying factors could lead to the deterioration of the disease and make the scenario worse. However, the impact of comorbidities on COVID-19 must be carefully considered.

In this analysis, patients had various symptoms but fever, cough, dyspnea, diarrhea, and shortness of breath were the most common clinical symptoms among patients with fungal co-infections and COVID-19. So far, similar results have been reported in this context (Singhal, 2020; Team, 2020). One study of 53 cases of HIV co-infection with COVID-19 indicated that fever, cough, and respiratory and gastrointestinal problems were the most common clinical symptoms reported in patients with SARS-CoV-2-HIV co-infection (Patel et al., 2021). In another study performed by Galván Casas et al. (2020) in Spain, the most common clinical symptoms among patients with COVID-19 were found to be fever, cough, pneumonia, vomiting, diarrhea, headache, nausea, and dyspnea.

As stated in the literature, concurrent involvement of various microorganisms in patients with COVID-19 is a serious threat, especially in patients with underlying diseases, which can lead to exacerbation of complications and subsequently increase the mortality rate. Infection with this virus is related to immune dysregulation, overexpression of pro-inflammatory cytokines, impaired cell-mediated immunity, and decreased CD4 and CD8^+^ T-cells that can increase the risk of invasive fungal infections (Hughes et al., 2020; Rawson et al., 2020; Farhan et al., 2021). However, there is scarce information regarding fungal co-infections and COVID-19. Therefore, adequate information is required on the simultaneous infection in patients with COVID-19 in adopting more appropriate treatment regimens for these patients. As it is well documented, patients with COVID-19 are at a greater risk of developing fungal infections because of its effect on the immune system and because treatments for COVID-19 can weaken the body’s defenses against fungi (Pemán et al., 2020; Rawson et al., 2020). According to the evidence, the number of reports of fungal co-infections in patients with COVID-19 was steadily growing worldwide. Awareness of the possibility of fungal co-infection with COVID-19 is essential to reduce delays in diagnosis and treatment in order to help prevent severe illness and death from these infections. In this analysis, infection with *Aspergillus* spp., *Mucor* spp., *Rhizopus* spp., *Candida* spp., and *P. jirovecii* was the most recorded fungal co-infections in patients with COVID-19. Similar findings of the main fungal co-infections in patients with COVID-19, such as *Aspergillus*, were also reported by studies conducted in China and Spain (Pemán et al., 2020; Song et al., 2020). In other study performed by Hoenigl (2020) and Garcia-Vidal et al. (2021), the most fungal infections in patients with COVID-19 include aspergillosis, invasive candidiasis, and mucormycosis. A study conducted by Chen et al. (2020) indicated a high prevalence of opportunistic fungal pathogens, such as *Aspergillus* spp., *Candida glabrata*, and *Candida albicans*, in patients with COVID-19. In this connection, Peng et al. (2021) in their systematic review and meta-analysis noted a 0.12 pooled proportion of fungal co-infection in patients with COVID-19. In a recent meta-analysis of eighteen studies, Rawson et al. (2020) reported that 8% of patients with COVID-19 had bacterial/fungal co-infection. The findings of this study indicated that the most COVID-19-associated mucormycosis is found in India. A study conducted in 2021 found that more than 47,000 cases of COVID-19-associated mucormycosis were reported in just 3 months in India (Muthu et al., 2021). Uncontrolled diabetes and overuse of steroids for COVID-19 treatment are important risk factors.

Geological differences have influenced the occurrences of fungal co-infection. Based on this meta-analysis, the frequency of fungal co-infection in patients with COVID-19 was higher in Asia than in other continents. Peng et al. (2021) reported that the fungal co-infection rate was significantly higher in patients from Asia than non-Asian patients.

The use of proper diagnostic techniques is an important issue in the management of COVID-19 diseases. CT scan is considered a relatively high sensitive method for diagnosing cases of COVID-19. This diagnostic method can be a useful factor for diagnosis and assessment of the infection progression in patients with COVID-19. However, the aforementioned technique may not find the involvement of the lung in the first stages of the disease and may not reliably confirm COVID-19 in the patients. According to the CT scan findings obtained from case reports and case series research, ground-glass opacification and bilateral infiltrates were reported as the predominant features in patients with fungal co-infections and COVID-19. This finding was similar to the findings of Radpour et al. (2020) and Omidi et al. (2021).

Diagnosing fungal co-infections in patients with COVID-19 is a serious challenge for clinicians, and it requires detection by a comprehensive diagnostic test for the achievement of an effective treatment. According to the analysis performed in this study, culture was the most common diagnostic method for the presence of fungal infections. As presented in the current analysis, the frequency of fungi in research using non-molecular assays is higher than in studies using molecular assays. As specified by Song et al. (2020), laboratory tests, including direct microscopic, culture, histopathology, BDG, real-time PCR, PCR, and MALDI-TOF techniques, can be used for the detection of fungal co-infections in patients with COVID-19. Since this laboratory evidence can alert us related to the severity of the disease, therefore, it is important to use these methods in combination for the diagnosis of fungal co-infections in these patients.

This study has some limitations. Since only case reports and case series studies have been selected for this review, they are more likely to be biased than other studies. Case studies and case series are descriptive and describe the patient’s signs and symptoms. The prevalence and percentage of co-infection in them have not been studied. For this reason, it was not possible to perform meta-analysis calculations in this review. Therefore, the prevalence of fungal infections among patients with COVID-19 has not been calculated.

## Conclusion

There have been many reported cases of viral, fungal, and bacterial infections associated with COVID-19. In this study, we studied the association between fungal infections and COVID-19. We discussed the clinical characteristics, diagnosis, treatment, and mortality rate of patients with COVID-19 co-infected with fungal infections. Sometimes the diagnosis of fungal infections occurs later in patients with COVID-19, which causes the progression and severity of the disease. Both diseases have similar risk factors, such as old age, diabetes, immunodeficiency, HIV, and COPD. Finally, a regular program is recommended to detect fungal infections during the outbreak of COVID-19 and follow it up continuously to prevent the occurrence of these two diseases simultaneously.

## Author Contributions

SS, MR-A, and MG designed the study. PB, MG, and MN performed the search, study selection, and data synthesis. SS and MG wrote the first draft of the manuscript. MN, MR-A, and SS revised the manuscript. All authors contributed to the article and approved the submitted version.

## Conflict of Interest

The authors declare that the research was conducted in the absence of any commercial or financial relationships that could be construed as a potential conflict of interest.

## Publisher’s Note

All claims expressed in this article are solely those of the authors and do not necessarily represent those of their affiliated organizations, or those of the publisher, the editors and the reviewers. Any product that may be evaluated in this article, or claim that may be made by its manufacturer, is not guaranteed or endorsed by the publisher.
